# Prevalence and Genetic Spectrum of Inherited Kidney Diseases: A Chinese Cohort Study

**DOI:** 10.34067/KID.0000001183

**Published:** 2026-04-01

**Authors:** Ziqi Liu, Jing Zhuang, Yan Yang, Ailima Aierken, Fengmei Wang, Zuolin Li, Qing Yin, Yan Tu, Hong Jiang, Bin Wang

**Affiliations:** 1 Department of Nephrology, Zhongda Hospital, Southeast University, Nanjing, Jiangsu, China; 2 Division of Nephrology, Department of Internal Medicine, People's Hospital of Xinjiang Uygur Autonomous Region, Urumqi, Xinjiang, China

**Keywords:** ADPKD, CKD, renal biopsy, genetic kidney disease

## Abstract

**Key Points:**

- A molecular genetic diagnosis was achieved in 31.4% of Chinese families.
- This study has firstly identified six genes as the principal causative genes underlying CKD in Chinese patients.

**Background:**

Inherited kidney disease (IKD) significantly contributes to CKD in children, adolescents, and adults. However, large-scale research on the prevalence of IKD in a Chinese population is currently lacking. To address this gap, we use exome sequencing to thoroughly investigate the prevalence and disease spectrum of IKD in a Chinese population.

**Methods:**

Exome sequencing was conducted on 290 patients with kidney disease of unknown etiology from two centers. Genetic test results were interpreted following the American College of Medical Genetics and Genomics guidelines and the criteria for variants of uncertain significance. Clinical data were integrated to establish a definitive diagnosis.

**Results:**

A total of 290 patients from 261 families were included in this study. Diagnostic variants were identified in 82 families, yielding a diagnostic rate of 31% (82/261). Six genes (*PKD1*, *PKD2*, *COL4A3*, *COL4A4*, *COL4A5*, and *UMOD*) accounted for 50% (41/82) of diagnosed cases. Ciliopathies were the most common subtype, followed by tubulopathies, collagenopathies, and podocytopathies. The diagnostic rate was higher in the age groups 20 years or younger and 41 years or older, at 63% and 39%, respectively. Among 51 biopsied patients, glomerular lesions (34/51) were the most common pathological type, followed by tubulointerstitial lesions (11/51). Of the 14 genetic diagnoses, 12 (86%) were consistent with histopathologic findings.

**Conclusions:**

A molecular genetic diagnosis was achieved in 31% of selected Chinese families. Genetic and clinical diagnosis complement each other, highlighting the application value of genetic testing in the diagnosis of IKD.

## Visual Abstract

**Figure:**
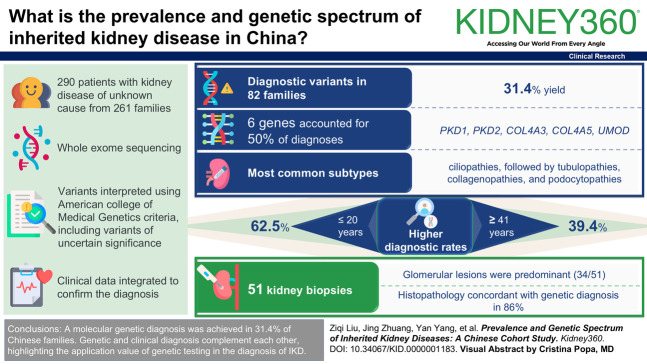

## Introduction

CKD is a progressive disease affecting approximately 800 million individuals worldwide. Epidemiological studies have shown that the prevalence of CKD is on a continuous upward trend. It is expected that by 2040, CKD will become the fifth leading cause of reduced life expectancy globally.^1^ The etiology of CKD is multifactorial and complex, with genetic factors significantly influencing disease progression. Research indicates that inherited kidney disease (IKD) constitutes a significant etiological component of CKD, affecting approximately one in every ten to 11 adults. A meta-analysis of 12 studies involving 6607 participants revealed that 22.2% of individuals carry diagnostic variants of monogenic kidney disease genes.^2^ IKD as the leading cause of ESKD in children and adolescents and the fifth leading cause of ESKD in adults has garnered substantial attention.^3^

The rapid development of next-generation sequencing (NGS) has brought revolutionary breakthroughs to the genetic research of kidney diseases. Specifically, exome sequencing (ES) and whole genome sequencing have significantly enhanced the diagnostic efficiency of IKD. A study has shown that NGS technologies can achieve a diagnosis rate of up to 48% in a select IKD cohort, as well as correct initial diagnoses of some patients.^4^

Currently, monogenic diseases account for 30%–50% of juvenile patients with CKD and 10%–20% of adult patients with CKD.^5–8^ To date, approximately 600 single genes have been identified as being associated with CKD.^9^ With the progress and development of sequencing technology, more kidney disease-associated genes are being discovered. Genetic testing not only aids in identifying the causes of unexplained kidney disorders but also allows for early diagnosis, prognosis evaluation, and the formulation of personalized treatment strategies. Furthermore, understanding the genetic basis of kidney disease is of great significance for risk assessment and genetic counseling for family members of patients. The Kidney Disease Improving Global Outcomes initiative recently highlighted the importance of genetics in the classification and management of CKD. It recommended that clinicians incorporate genetic testing into routine procedures to improve diagnostic accuracy and promote precision nephrology. Through genetic testing, clinicians can acquire a better understanding of the genetic foundations of the disease and optimize treatment plans.^10^

In this study, 290 patients with unexplained kidney disease or clinically suspected IKD were included for ES. It aims to explore the genetic characteristics of IKD in a Chinese multicenter cohort, clarify its prevalence, and analyze the distribution of the disease between Han and non-Han ethnic groups, as well as the genetic etiologies identified in this cohort. In addition, this study will evaluate the clinical application value of genetic testing in the diagnosis and treatment of IKD and further explore its utility in clinical inherited nephrology and its potential in enhancing patient diagnosis and treatment.

## Methods

### Patient Population

A retrospective cohort study was conducted, identifying 310 patients with CKD who were hospitalized in Zhongda Hospital Southeast University and People's Hospital of Xinjiang Uygur Autonomous Region from November 2018 to September 2024. A total of 290 patients who met the criteria were ultimately included, among whom 51 patients underwent kidney biopsy. The clinical diagnostic classification of CKD is as follows: vascular nephropathy, glomerular nephropathy, tubulointerstitial nephropathy, congenital anomalies of the kidney and urinary tract (CAKUT) or cystic nephropathy, and unexplained nephropathy.

Inclusion criteria were as follows: (*1*) patients with CKD of unknown etiology; (*2*) patients with clinical manifestations of hereditary diseases, such as multiple organ involvement, congenital anomalies, age of onset 30 years or younger,^11^ as well as consanguineous marriages or family history of kidney disease; and (*3*) pretransplant screening.

Exclusion criteria were as follows: Individuals with a definitive diagnosis of nongenetic kidney disease, such as diabetic nephropathy, hypertensive nephropathy, or secondary kidney diseases (*e.g*., SLE, vasculitis), were excluded.

Signed informed consent was obtained from all patients and/or their parents. This study was approved by the Clinical Research Ethics Committee of the Affiliated Zhongda Hospital of Southeast University.

### Clinical Data Collection

We analyzed electronic medical records to collect thorough patient data, which included age, sex, age of onset, clinical symptoms, extrarenal characteristics, and a detailed family history. We gathered and examined histopathological findings found in the biopsy specimens of 51 patients who had undergone kidney biopsy. Furthermore, we documented the genetic testing results, amino acid changes, nucleotide alterations, genomic position, and *de novo* variants.

### Statistical Analysis

Statistical analyses were performed using SPSS Statistics 27 software. The baseline characteristics were reported as frequency (*n*, %), mean, SD, and median (range). For continuous variables, normality tests were run on continuous variables initially. Normally distributed continuous variables are expressed as (x̄±s), and the independent sample *t* test was used for comparisons between the two groups. Non-normally distributed continuous variables are expressed as M (P25, P75), and the rank-sum test was used for comparisons between the two groups. Categorical variables are expressed as counts and percentages, and the chi-squared test was used for comparisons between the two groups.

### Variant Analysis

**Figure 1:**
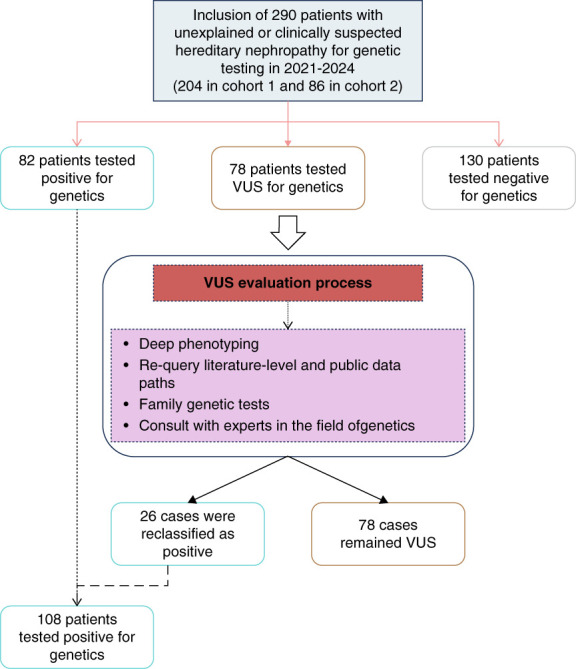
**Diagnostic workflow for unexplained nephropathy or clinical suspicion of hereditary nephropathy.** Cohort 1: Zhongda Hospital Southeast University; Cohort 2: People's Hospital of Xinjiang Uygur Autonomous Region. VUS, variants of uncertain significance.

Sequence variants were interpreted following the American College of Medical Genetics and Genomics (ACMG) standards and guidelines, with refinements from the ClinGen Sequence Variant Interpretation Working Group. For novel variants not previously reported in public databases (gnomAD, ClinVar) or peer-reviewed literature, the following criteria were systematically applied: (*1*) population frequency assessment (PM2) using a threshold of <0.0001 for autosomal dominant disorders and <1% for recessive disorders; (*2*) computational predictions (PP3) using rare exome variant ensemble learner score ≥0.7 for missense variants and SpliceAI ≥0.2 for splicing variants; (*3*) loss-of-function variant evaluation according to the ClinGen loss-of-function variant evaluation decision tree, with strength adjustment when functional confirmation was unavailable; (*4*) *de novo* status (PS2/PM6) on the basis of parental confirmation; and (*5*) cosegregation analysis (PP1) when family members were available. Variants were classified as pathogenic, likely pathogenic, variant of uncertain significance (VUS), likely benign, or benign using standard ACMG combination rules. For patients initially diagnosed with VUS who underwent reclassification, in-depth phenotypic analysis, literature review, family segregation studies, and expert consultation were conducted as described in Figure 1.

Gene detection positivity was determined by classifying variants as pathogenic or likely pathogenic, and the diagnostic rate was calculated for Han and non-Han ethnic groups, including those who had their VUS re-evaluated as gene detection positive. *P* values <0.05 were considered statistically significant.

## Results

### Baseline Data

A total of 290 patients were included, comprising 204 patients from Zhongda Hospital Southeast University (82 female patients) and 86 patients from the People's Hospital of Xinjiang Uygur Autonomous Region (26 female patients). The median age was 42 (28–53) years. Glomerular nephropathy (22%) was the most common clinical subtype suspected of being IKD. Eighty-eight individuals (30%) had a family history of kidney disease. According to the clinical diagnostic classification, the disease spectrum was distributed as follows: CAKUT or cystic nephropathy in 25 cases (28%), glomerular nephropathy in 17 cases (19%), vascular nephropathy in four cases (5%), tubulointerstitial nephropathy in two cases (2%), and CKD of unknown etiology in 40 cases (46%; Supplemental Figure 1). Extrarenal manifestations were present in 149 patients (51%). Kidney biopsy was performed in 18% of the cohort (*n*=51), with FSGS identified as the most prevalent pathological subtype. The distribution of CKD stage at the time of ES was as follows: stages 1–2 (*n*=55), stages 3–4 (*n*=37), and stage 5 (*n*=78) (Table 1).

**Table 1:** Patients baseline characteristics

Characteristic	Whole Cohort (*N*=290)	Cohort 1 (*n*=204)	Cohort 2 (*n*=86)
Age at CKD onset	34 [25–47]	38.5 [27–51]	31 [24–43]
Age at time of study entry (yr)	42 [28–53]	43 [28–55]	36 [28–47]
Sex—female, no. (%)	108 (37)	82 (40)	26 (30)
Nationality—Han (%)	254 (88)	204 (100)	50 (58)
Family history of kidney disease, no. (%)	88 (30)	73 (36)	15 (17)
**Clinical diagnosis, no. (%)**			
CAKUT or cystic	60 (21)	48 (24)	12 (14)
Glomerular	63 (22)	50 (25)	13 (15)
Tubulointerstitial	13 (5)	11 (5)	2 (2)
Vascular	9 (3)	8 (4)	1 (1)
CKD etiology unknown	145 (50)	87 (43)	58 (67)
Extrarenal manifestations, no. (%)	149 (51)	107 (53)	42 (49)
Patient with kidney biopsy, no. (%)	51 (18)	39 (19)	12 (14)
Glomerular lesions	34	25	9
Vascular lesions	1	1	0
Tubulointerstitial lesions	11	9	2
Nonspecific lesions	5	4	1
**Stage of kidney disease at ES (%)**			
1–2	55 (19)	55 (27)	
3–4	37 (13)	36 (18)	1 (1)
5	78 (27)	48 (24)	30 (35)

Cohort 1, Zhongda Hospital Southeast University; Cohort 2, People's Hospital of Xinjiang Uygur Autonomous Region; continuous values are presented as median with interquartile range. Categorical values are presented as number (%). CAKUT, congenital anomalies of the kidney and urinary tract; ES, exome sequencing.

### Genetic Testing Results

#### Positive Diagnoses and VUS Re-Evaluation Results from ES Testing

The study included 290 patients from 261 different families, with a total of 108 positive diagnoses (Table 2). Pathogenic gene variants were detected in 82 families, with a diagnostic rate of 31% (82/261). For the 52 patients diagnosed with VUS, re-evaluation was conducted through in-depth phenotypic analysis, re-querying existing literature and public databases, family genetic testing, and consulting with genetics experts. As a result, 26 resolved VUS patients were reclassified as positive diagnosis (shown in Figure 1). Patients with extrarenal manifestations (73% versus 32%, *P* < 0.001) and a family history of kidney disease (44% versus 22%, *P* < 0.001) had higher positive diagnostic rates in genetic testing. There are no differences between two groups in age, sex, age of onset, nationality, and stage of kidney disease at the time of ES.

**Table 2:** Comparison of characteristics between patients with positive and negative genetic test results

Characteristic	Patients with a Positive Genetic Diagnosis (*n*=108)	Patients with a Negative Genetic Diagnosis (*n*=130)	*P* Value
Age at CKD onset	34 [24–51]	37 [25–48]	0.97
Age at time of study entry (yr)	43 [29–54]	43 [29–54]	0.83
Sex—female, no. (%)	45 (42)	46 (35)	0.32
**Nationality (%)**			0.06
Han	100 (93)	110 (85)	
Other	8 (7)	20 (15)	
Family history of kidney disease, no. (%)	47 (44)	28 (22)	<0.001
**Clinical diagnosis, no. (%)**			<0.001
CAKUT or cystic	38 (35)	19 (15)	
Glomerular	20 (19)	27 (21)	
Tubulointerstitial	9 (8)	3 (2)	
Vascular	7 (7)	2 (2)	
CKD etiology unknown	34 (32)	79 (61)	
Extrarenal manifestations, no. (%)	79 (73)	36 (28)	<0.001
**Patient with kidney biopsy, no. (%)**	14 (13)	27 (21)	0.11
Glomerular lesions	10	16	
Vascular lesions	1	0	
Tubulointerstitial lesions	3	7	
Nonspecific lesions	0	4	
**Stage of kidney disease at ES (%)**			0.95
1–2	18 (17)	26 (20)	
3–4	12 (11)	17 (13)	
5	29 (27)	34 (26)	

Continuous values are presented as median with interquartile range. Categorical values are presented as number (%). CAKUT, congenital anomalies of the kidney and urinary tract; ES, exome sequencing.

#### Disease Distribution and Characteristics of Novel Variants in Positive Patients

**Figure 2:**
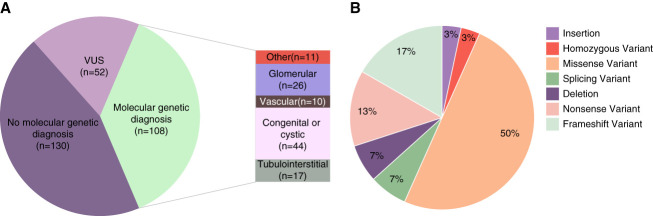
**Distribution of IKDs and new variant types of positive patients.** (A) The molecular diagnostic results of 290 patients after ES and VUS evaluation process. A positive molecular genetic diagnosis was established in 108 patients, indicated in green; VUS were detected in 52 patients in known CKD genes, indicated in purple; dark purple indicates that 130 patients did not have a molecular genetic diagnosis established after ES. (B) The type and percentage of new variants in 35 positive patients. ES, exome sequencing; IKD, inherited kidney diseases.

Among the 108 patients with confirmed genetic diagnoses, the spectrum of IKD was distributed as follows: CAKUT or cystic kidney diseases had the highest proportion, accounting for 41% (44/108), followed by glomerular diseases at 24% (26/108), tubulointerstitial diseases at 16% (17/108), and thrombotic microangiopathies at 9% (10/108; shown in Figure 2A).

A total of 35 *de novo* pathogenic variant sites were identified in this study (Supplemental Table 1). Among them (Supplemental Table 2), missense variants had the highest proportion, reaching 49% (17/35), followed by frameshift and splicing variants, which accounted for 26% (9/35) and 14% (5/35), respectively (shown in Figure 2B).

**Figure 3:**
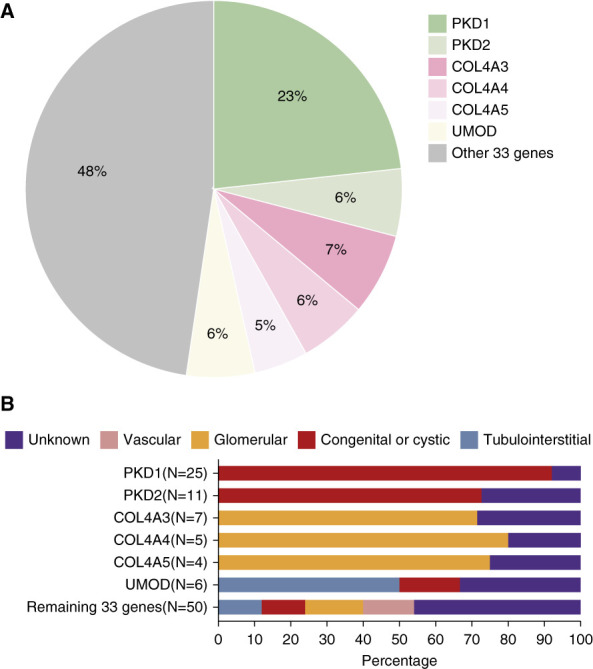
**Positive gene discovery and clinical diagnosis spectrums.** (A) Distribution of positive genes and common genes. (B) The clinical diagnostic spectrum of positive genes. Note: The *x* axis shows the percentage of various types of clinical diagnoses; the observed clinical diagnostic spectrum of the other 33 genes is shown next to it for comparison. The clinical diagnostic categories were CAKUT or cystic nephropathy, glomerular nephropathy, tubulointerstitial nephropathy, vascular nephropathy, and unexplained nephropathy. CAKUT, congenital anomalies of the kidney and urinary tract.

In the 108 diagnoses of IKD, a total of 39 distinct monogenic disorders were identified. One patient presented with a dual molecular diagnosis (compound heterozygous variants in *CFB* and *COL4A1*). Six pivotal genes (*PKD1*, *PKD2*, *COL4A3*, *COL4A4*, *COL4A5*, and *UMOD*) contributed to 52% of the genetic diagnoses (shown in Figure 3A). Notably, patients with *UMOD*-associated tubulointerstitial disease exhibited significant clinical heterogeneity (shown in Figure 3B). On the basis of molecular pathogenesis, the 39 monogenic diseases were classified, and the result showed that ciliopathies were the predominant type of IKD, accounting for 38% of the total diagnosed cases (*PKD1* [*n*=25], *PKD2* [*n*=11], *PKHD1* [*n*=2], *DNAJB11* [*n*=1], *GANAB* [*n*=1], nephronophthisis *3* [*n*=1]), followed by tubulopathies with a percentage of 20% (*UMOD* [*n*=6], *CUBN* [*n*=4], *ATP6V0A4* [*n*=3], *SLC12A3* [*n*=3], *CLCNKB* [*n*=2], *KCNJ16* [*n*=1], *SCNN1B* [*n*=1], *WDR72* [*n*=1]). Collagenopathies (*COL4A3* [*n*=8], *COL4A4* [*n*=4], *COL4A5* [*n*=4]) and podocytopathies (*WT1* [*n*=3], *PLCE1* [*n*=2], *DAAM2* [*n*=1], *NPHS1* [*n*=1], *PAX2* [*n*=1], *PIGA* [*n*=1]) were the third and fourth most common hereditary nephropathy categories, accounting for 15% and 8%, respectively (shown in Figure 5).

**Figure 4:**
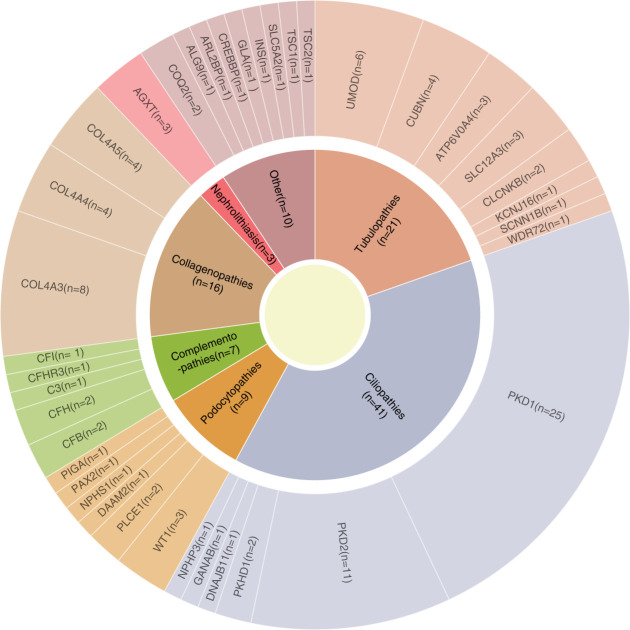
**Distribution of genes for IKDs.** Inner circle represents the category of IKDs; outer circle represents positive genes reported in patients; numbers in parentheses represent the number of carriers. NPHP, nephronophthisis.

**Figure 5:**
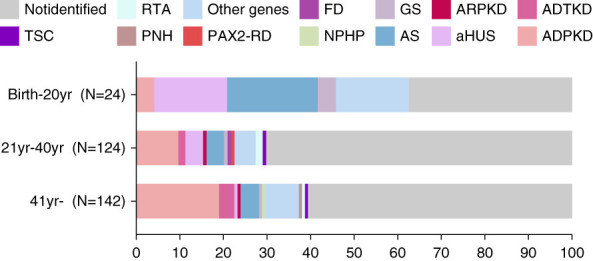
**Description of the disease according to age group at the time of genetic testing.** ADPKD, autosomal dominant polycystic kidney disease; ADTKD, autosomal dominant tubulointerstitial kidney disease; aHUS, atypical hemolytic uremic syndrome; ARPKD, autosomal recessive polycystic kidney disease; AS, Alport syndrome; FD, Fabry disease; GS, Gitelman syndrome; PNH, paroxysmal nocturnal hemoglobinuria; RTA, renal tubular acidosis; TSC, tuberous sclerosis complex.

**Figure 6:**
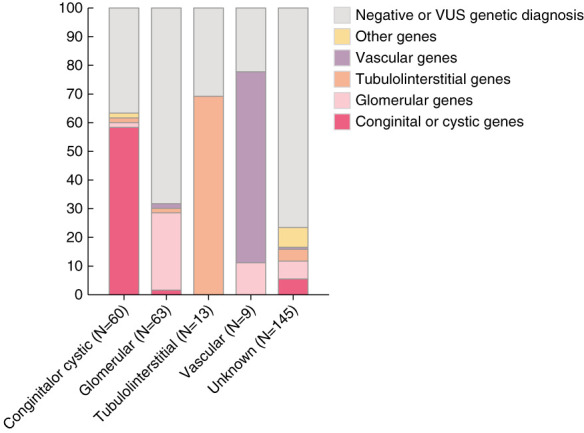
**Rates of genetic diagnosis in each clinical diagnostic group.** The *x* axis shows the clinical diagnosis of 290 patients before genetic testing; the *y* axis represents the genetic diagnosis after genetic testing. Patients were divided into five groups: CAKUT or cystic nephropathy-related genes (pink); glomerular disease-related genes (light pink); tubulointerstitial nephropathy-related genes (orange); vascular genes (purple); and after ES, no molecular genetic diagnosis is established (gray).

Age-stratified analysis of IKD is presented in Figure 5. The diagnostic rates of IKD were relatively higher in the age groups of 20 years or younger and 41 years or older, at 63% and 39%, respectively. Alport syndrome was detected across all age groups, with a particularly significant proportion in the 20 years or younger cohort. The incidence of genetic variants associated with atypical hemolytic uremic syndrome was substantially greater in the 20 years or younger group compared with other groups. By contrast, autosomal dominant polycystic kidney disease had the highest proportion in the 41 years or older group, reaching 19% (Figure 6).

### Genetic Diagnosis and Histopathologic Findings

The pathological results of kidney biopsy specimens revealed that the most common histopathological finding was a glomerular lesion (34/51), followed by tubulointerstitial lesions (11/51) and nonspecific histologic lesions (5/51), with vascular lesions in one patient (1/51). Among the 14 genetic diagnoses, 12 (86%) were consistent with the histopathological results. The top three groups with the highest diagnostic rates of ES were glomerular disease, tubulointerstitial disease, and thrombotic microangiopathy, with diagnostic rates of 50% (7/14), 29% (4/14), and 14% (2/14), respectively (Table 2).

### Analysis of Genetic Diagnosis Rates in Different Clinical Diagnostic Groups

Among the 108 patients with positive genetic diagnoses, 53 (49%) experienced diagnostic delays, with an average diagnostic delay of 10.7 years. Notably, 21 (19%) patients did not receive a definitive diagnosis until they began dialysis.

Significant disparities in genetic diagnostic rates were observed across different clinical diagnostic groups in this study: The genetic diagnostic rate was 63% for CAKUT or cystic nephropathy, 32% for glomerulopathy, 62% for tubulointerstitial disease, and 78% for vascular nephropathy. Furthermore, ES provided molecular diagnoses for 23% of families that were clinically classified as having kidney disease of unknown etiology (Figure 6).

## Discussion

ES was used to systematically investigate IKD in the Chinese population. The findings revealed that 31% of families received a definitive molecular diagnosis through genetic testing. CAKUT or cystic nephropathy emerged as the most prevalent IKD subtype. The genes most commonly associated with CKD in this cohort were *PKD1*, *PKD2*, *COL4A3*, *COL4A4*, *COL4A5*, and *UMOD*. This study is the first to construct a molecular diagnostic profile of IKD specifically based on a mainland Chinese cohort and to estimate its incidence within this specific population. These findings enhance clinicians’ understanding of the genetic landscape and clinical features of IKD, ultimately aiding in improved diagnosis and management.

An ES study of 3315 adult patients with CKD identified *PKD1*, *PKD2*, *COL4A3*, *COL4A4*, *COL4A5*, and *UMOD* as the most frequently implicated genes in monogenic CKD,^7^ which is highly consistent with the findings of the present investigation. Similarly, Bleyer *et al.* conducted NGS on 1007 patients and confirmed the critical roles of *PKD1*, *COL4A5*, *PKD2*, *COL4A4*, and *COL4A3*.^12^ Notably, despite differences in inclusion criteria (this study focused on patients with CKD of unknown etiology or those clinically suspected of having IKD), and detection methods (ES was employed here), the five genes—*PKD1*, *PKD2*, *COL4A3*, *COL4A4*, *COL4A5*—consistently demonstrated significant pathogenic associations. In this study, these five genes accounted for over half of the molecular diagnoses in 82 confirmed families, underscoring their central role in the genetic etiology of monogenic CKD.

Our investigation found that ciliopathies were the most common disease category among IKDs, whereas Robert *et al.*,^13,14^ using ES, identified collagenopathies as the predominant genetic category in patients with undiagnosed kidney disease (shown in Figure 4). This discrepancy may be attributed to differences in study design: This study was not limited to patients with undiagnosed kidney disease but included a broader cohort of individuals clinically suspected to have IKD, notably encompassing polycystic kidney disease—a major subtype of ciliopathies. Regarding age distribution, this study revealed that Alport syndrome (a collagen-related disease) was the most prevalent diagnosis in patients 20 years or younger (63%), while autosomal dominant polycystic kidney disease, a ciliopathy, was most common in patients 41 years or older (39%). These findings align with the natural progression of inherited nephropathies and are consistent with existing epidemiological data, illustrating the age-related distribution patterns of hereditary kidney disorders (shown in Figure 5).

The influence of sex on diagnostic yield varies across studies. Thomas^14^ and Connaughton^15^ observed higher diagnostic rates in female patients with X-linked conditions, whereas Becherucci *et al.*,^16^ in a cohort of 392 patients with suspected IKD, found no significant difference in diagnostic yield by sex (*P* = 0.32). These discrepancies may reflect variations in the disease spectrum across different studies. Importantly, female carriers of X-linked disorders may exhibit milder clinical symptoms yet experience a prolonged disease course. These considerations highlight the need to account for sex as a factor in clinical practice: (*1*) long-term follow-up is recommended for female patients; (*2*) additional monitoring should be implemented during pregnancy; and (*3*) sex-specific guidance should be incorporated into genetic counseling. Future research should include sex-stratified analyses to further elucidate the role of sex in the diagnosis and management of IKD.^17^

Genetic cosegregation analysis plays a pivotal role in evaluating the pathogenicity of gene variants. Within affected families, multiple phenotypes are assessed through clinical re-evaluation and segregation analysis. According to the ACMG guidelines, novel cosegregation observed in patients with VUS can support reclassification of these variants as pathogenic.^18^ However, ES performed solely on probands may lead to missed diagnoses. By contrast, family-based ES can partially mitigate this limitation by providing additional inheritance information. In this study, strict adherence to ACMG guidelines enabled the reclassification of 26 patients initially diagnosed with VUS into positive molecular diagnoses by integrating clinical re-evaluation with family-based segregation analysis. Notably, in one case, family-based ES of a patient with an initial missed diagnosis revealed dual pathogenic variants in *CFB* and *COL4A5*.^19^ These findings underscore the importance of interpreting genetic test results in the context of patient phenotype, inheritance patterns, and established clinical guidelines. Nephrologists must adopt a comprehensive approach when evaluating patients with suspected IKD.

Although kidney biopsy is a commonly used method for identifying the etiology of kidney disease, it is not suitable for all patients. In this study, an 86% concordance was observed between genetic diagnoses and histopathological findings, indicating that these two diagnostic modalities are highly complementary. Genetic testing can address the limitations of conventional pathological diagnosis, especially in early-stage disease or atypical clinical presentations, and can support more precise treatment planning in later stages. It also facilitates subsequent precision treatment, demonstrating high clinical value. For example, in patients with atypical hemolytic uremic syndrome who have complement gene mutations or positive antibodies, eculizumab can specifically inhibit the terminal complement pathway, rapidly block microvascular thrombosis, and preserve renal function. Genetic testing results are the key basis for determining whether to initiate this expensive but highly effective treatment.^20^ In our study, a patient hospitalized for proteinuria presented kidney biopsy findings suggestive of IgA nephropathy alongside myeloid cells consistent with lysosomal storage disease. Genetic testing ruled out *GLA* gene variants, preventing misdiagnosis. The same test identified a novel pathogenic mutation in the *COQ2* gene,^21^ establishing the patient's core diagnosis. Initial prednisone treatment exacerbated proteinuria. After genetic testing, therapy was switched to coenzyme Q10 supplementation targeting the underlying genetic cause. Two months later, proteinuria significantly decreased. Genetic testing successfully shifted the treatment strategy from ineffective immunosuppression to effective metabolic supplementation therapy, achieving precision medicine. Beyond reducing the risks associated with kidney biopsy—including bleeding, hematoma, hematuria, and, in rare cases, death^22^—genetic testing offers several clinical advantages. These include facilitating the selection of optimal kidney donors for patients with ESKD, enabling screening and management of extrarenal manifestations, guiding reproductive decision-making, and informing post-transplantation care strategies.^23^

This study partially reveals the genetic characteristics of IKD in the Chinese population but also presents several limitations. First, the detection technology has certain constraints: Although ES is highly sensitive for diagnosing IKD, it does not capture noncoding region variants, structural variants, or copy number variants, which could result in the omission of disease-causing genes.^24^ In addition, the detection efficiency of ES may be reduced for highly repetitive sequences or regions with high glomerular capillaries content. Second, the sample representativeness is limited: Among the 290 patients included, only 34 were non-Han population (12%). This uneven distribution could affect the accurate assessment of genetic traits in ethnic minorities. While our cohort is sufficiently large to characterize the prevalent genetic architecture of IKD, its size limits the ability to assess the contribution of variants that are extremely rare in the population, which may be absent by chance alone. Future studies should expand the sample size, particularly by increasing the representation of ethnic minority populations. They should also combine technologies, such as whole genome sequencing or targeted long-read sequencing, to improve the detection of noncoding region variants and structural variants. Establishing a multicenter collaborative network would also enhance the integration and analysis of clinical and genetic data.

In conclusion, this systematic multicenter cohort study has, for the first time, identified *PKD1*, *PKD2*, *COL4A3*, *COL4A4*, *COL4A5*, and *UMOD* as the principal causative genes underlying CKD in our cohort of Chinese patients. Genetic testing achieved a diagnostic yield of 31% among patients with kidney disease of unknown etiology, particularly in those with early-onset disease, a family history, extrarenal manifestations, or atypical pathological findings. Moreover, genetic analysis corrected initial clinical diagnoses in several cases, providing a molecular basis for the development of personalized treatment strategies. Future research should aim to increase cohort size—particularly among ethnic minority groups—and incorporate multiomics approaches to further advance precision diagnostics and therapeutic planning for IKD in the Chinese population.

## Supplementary Material

## Data Availability

All original data, including deidentified patient-level data or individual laboratory data measurements, are included in the manuscript and/or supplemental material.

## Declarative Statements

This study includes clinical experimentation and received Institutional Review Board or Ethics Committee approval. All patients provided written informed consent. This study includes clinical experimentation and complies with the Declaration of Helsinki. The clinical and research activities being reported are consistent with the Principles of the Declaration of Istanbul as outlined in the Declaration of Istanbul on Organ Trafficking and Transplant Tourism.

## Supplemental Material

Supplemental Figure 1. Clinical diagnosis spectrum of 88 patients with a family history of kidney disease.

Supplemental Table 1. Molecular genetic diagnostic information for 108 positive patients.

Supplemental Table 2. Variation frequency of each variant within the cohort and the variation frequency data retrieved from mainstream databases.

## References

[1] Bikbov B Purcell CA Levey AS, . Global, regional, and national burden of chronic kidney disease, 1990-2017: a systematic analysis for the global burden of disease study 2017. Lancet. 2020;395(10225):709–733. doi:10.1016/S0140-6736(20)30045-3 32061315 PMC7049905

[2] Eble J Köttgen A Schultheiß UT. Monogenic kidney diseases in adults with chronic kidney disease (CKD). Dtsch Arztebl Int. 2024;121(21):689–695. doi:10.3238/arztebl.m2024.0120 38958599 PMC12005384

[3] Zhang L Yue C Liu Y Chen LM. Rare genetic kidney diseases: windows of precision nephrology. J Rare Dis. 2024;3(1):1–11.doi:10.12376/j.issn.2097-0501.2024.01.001

[4] Topak A. Molecular diagnostic results of a nephropathy gene panel in patients with suspected hereditary kidney disease. Lab Med. 2024;55(1):13–19. doi:10.1093/labmed/lmad027 37078890

[5] Groopman EE Marasa M Cameron-Christie S, . Diagnostic utility of exome sequencing for kidney disease. N Engl J Med. 2019;380(2):142–151. doi:10.1056/NEJMoa1806891 30586318 PMC6510541

[6] Connaughton DM Kennedy C Shril S, . Monogenic causes of chronic kidney disease in adults. Kidney Int. 2019;95(4):914–928. doi:10.1016/j.kint.2018.10.031 30773290 PMC6431580

[7] Connaughton DM Hildebrandt F. Personalized medicine in chronic kidney disease by detection of monogenic mutations. Nephrol Dial Transplant. 2020;35(3):390–397. doi:10.1093/ndt/gfz028 30809662 PMC7057531

[8] Ottlewski I Münch J Wagner T, . Value of renal gene panel diagnostics in adults waiting for kidney transplantation due to undetermined end-stage renal disease. Kidney Int. 2019;96(1):222–230. doi:10.1016/j.kint.2019.01.038 31027891

[9] Kil SY Yang WS Kim YN Shin HS Jung Y Rim H. Genes, kidneys, and the future: transforming chronic kidney disease management through genomic insights. Child Kidney Dis. 2025;29(2):39–45. doi:10.3339/cd.25.016 PMC13587183 42779587

[10] Vivante A. Genetics of chronic kidney disease. N Engl J Med. 2024;391(7):627–639. doi:10.1056/NEJMra2308577 39141855

[11] Domingo-Gallego A Pybus M Bullich G, . Clinical utility of genetic testing in early-onset kidney disease: seven genes are the main players. Nephrol Dial Transplant. 2022;37(4):687–696. doi:10.1093/ndt/gfab019 33532864

[12] Bleyer AJ Westemeyer M Xie J, . Genetic etiologies for chronic kidney disease revealed through next-generation renal gene panel. Am J Nephrol. 2022;53(4):297–306. doi:10.1159/000522226 35325889 PMC9216312

[13] Robert T Greillier S Torrents J, Diagnosis of kidney diseases of unknown etiology through biopsy-genetic analysis. Kidney Int Rep. 2023;8(10):2077–2087. doi:10.1016/j.ekir.2023.07.003 37850010 PMC10577324

[14] Robert T Raymond L Dancer M, . Beyond the kidney biopsy: genomic approach to undetermined kidney diseases. Clin Kidney J. 2024;17(1):sfad099. doi:10.1093/ckj/sfad099 38186885 PMC10765093

[15] Connaughton DM Bukhari S Conlon P, . The Irish kidney gene project--prevalence of family history in patients with kidney disease in Ireland. Nephron. 2015;130(4):293–301. doi:10.1159/000436983 26202451

[16] Becherucci F Landini S Palazzo V, . A clinical workflow for cost-saving high-rate diagnosis of genetic kidney diseases. J Am Soc Nephrol. 2023;34(4):706–720. doi:10.1681/ASN.0000000000000076 36753701 PMC10103218

[17] Jankowska M Soler MJ Stevens KI Torra R. Why do we keep ignoring sex in kidney disease? Clin Kidney J. 2023;16(12):2327–2335. doi:10.1093/ckj/sfad183 38046033 PMC10689162

[18] Doreille A Lombardi Y Dancer M, . Exome-first strategy in adult patients with CKD: a cohort study. Kidney Int Rep. 2023;8(3):596–605. doi:10.1016/j.ekir.2022.12.007 36938085 PMC10014383

[19] Wang FM Yang Y Zhang XL, . Combination of a novel genetic variant in CFB gene and a pathogenic variant in COL4A5 gene in a sibling renal disease: a case report. Front Genet. 2021;12:690952. doi:10.3389/fgene.2021.690952 34349783 PMC8326751

[20] Walsh PR Kavanagh D. Atypical hemolytic uremic syndrome. J Allergy Clin Immunol. 2025;156(2):224–236. doi:10.1016/j.jaci.2025.05.010 40436118

[21] Ni HF Yang Y Li CQ Zhou TZ Liu BC Wang B. Myeloid bodies caused by COQ2 mutation: a case of concurrent COQ2 nephropathy and IgA nephropathy. Clin Kidney J. 2021;14(6):1697–1700. doi:10.1093/ckj/sfab043 34084467 PMC8162858

[22] Poggio ED McClelland RL Blank KN, . Systematic review and meta-analysis of native kidney biopsy complications. Clin J Am Soc Nephrol. 2020;15(11):1595–1602. doi:10.2215/CJN.04710420 33060160 PMC7646247

[23] Ma BM Elefant N Tedesco M, . Developing a genetic testing panel for evaluation of morbidities in kidney transplant recipients. Kidney Int. 2024;106(1):115–125. doi:10.1016/j.kint.2024.02.021 38521406 PMC11410071

[24] Zuk O Schaffner SF Samocha K, . Searching for missing heritability: designing rare variant association studies. Proc Natl Acad Sci U S A. 2014;111(4):E455–E464. doi:10.1073/pnas.1322563111 24443550 PMC3910587

