# Supplementary material for: Prevalence and Genetic Spectrum of Inherited Kidney Diseases: A Chinese Cohort Study
Source: Kidney360. 2026 Apr 1;7(7):1615–24. doi: 10.34067/KID.0000001183 (PMC13450970; doi:10.34067/KID.0000001183)
Supplement: Supplementary file 1 [file kidney360-7-1615-s001.pdf]

Supplemental Figure 1.Clinical diagnosis spectrum of 88 patients with a family history of renal disease

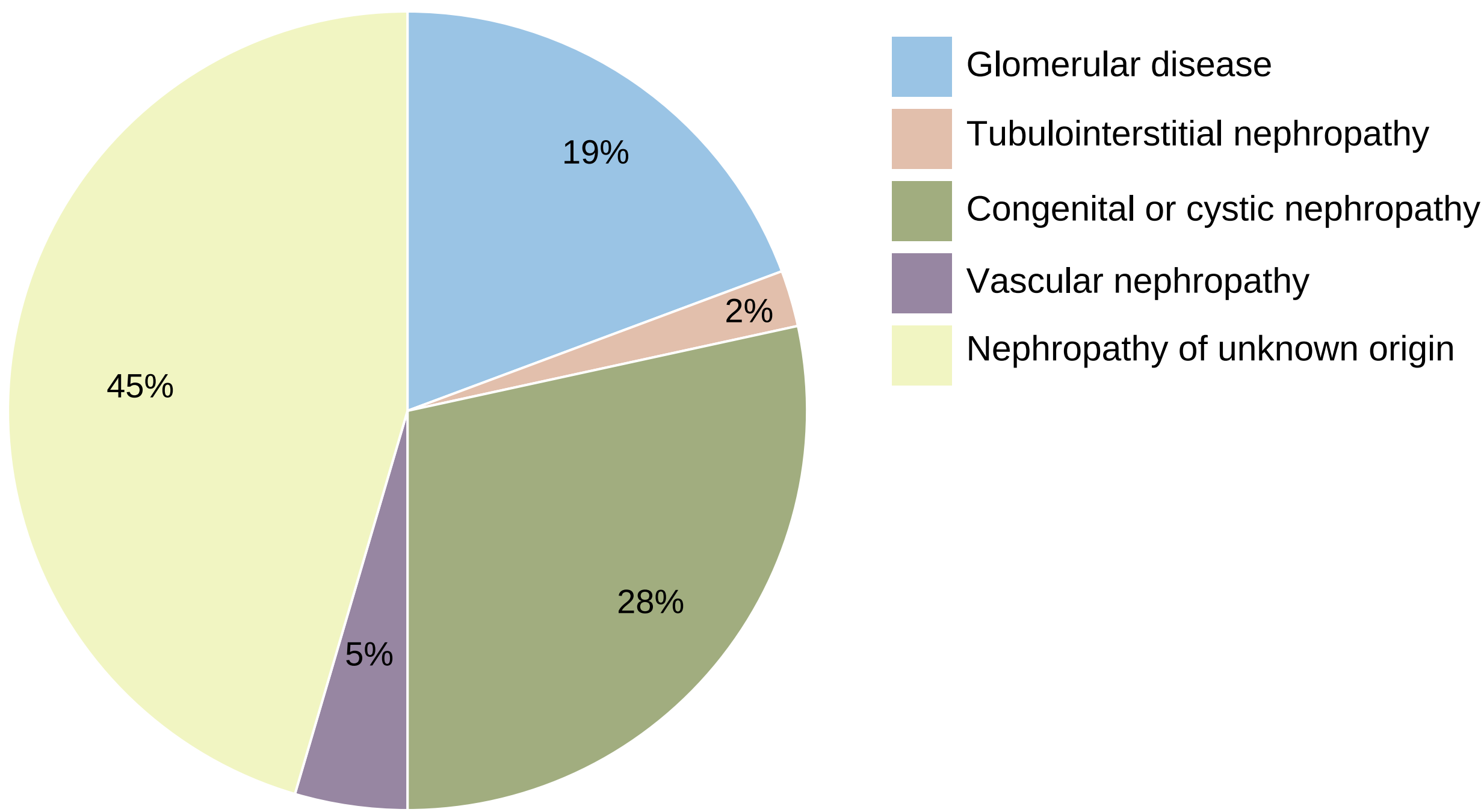

Supplemental Table 1: Molecular Genetic Diagnostic Information for 108 Positive Patients.

Patient	Gene	HGVS		ACMG Class	Chromosome	Absolute Position	Ref	Alt	Allele Count	Allele Number	Number of Homozygotes	Transcript	Genetic Type	Zygote Type
1	GLA	c.1033_1034delTC	p.Ser345Argfs*29	Likely pathogenic	chrX	100653052	TGA	T	-	-	-	NM_000169.3	XL	hem
2	PKD1#	c.6915_6915+2delGGT	-	Likely pathogenic	chr16	2158250	GACC	G	-	-	-	NM_001009944.3	AD	het
3	KCNJ16	c.689G>A	p.Arg230Lys	VUS	chr17	68128917	G	Alt	-	-	-	NM_018658.4	AR	het
4	AGXT	c.823_824dupAG	p.Ser275Argfs*38	Pathogenic	chr2	241815390	T	TGA	3	18392	0	NM_000030.3	AR	het
	AGXT	c.638C>T	p.Ala213Val	VUS	chr2	241813437	C	T	5	18382	0	NM_000030.3	AR	het
5	CFB#	c.121G>C	p.Gly41Arg	VUS	chr6	31914206	G	C	-	-	-	NM_001710.6	AD	het
6	CFB#	c.121G>C	p.Gly41Arg	VUS	chr6	31914206	G	C	-	-	-	NM_001710.6	AD	het
7	CFHR3#	c.613+90A>C	-	VUS	chr1	196757618	A	C	1	1540	0	NM_021023.6	AD, AR	het
8	CLCNKB	c.280C>A	p.Leu94Ile	VUS	chr1	16373080	C	A	83	19952	0	NM_000085.5	AR	het
9	COL4A3	c.1909G>A	p.Gly637Arg	VUS	chr2	228137815	G	A	1	17934	0	NM_000091.5	AD, AR	het
10	COL4A4#	c.1786G>T	p.Gly596Trp	VUS	chr2	227945176	C	A	-	-	-	NM_000092.5	AR	het
11	COL4A5	c.2215C>G	p.Pro739Ala	VUS	chrX	107846262	C	G	171	13249	0	NM_000495.5	XL	hem
12	NPHS1	c.3478C>T	p.Arg1160*	Pathogenic	chr19	36321958	G	A	1	18394	0	NM_004646.4	AR	het

13	PKD1	c.1997C>G	p.Ala666Gly	VUS	chr16	2165479	G	C	1	1560	0	NM_000296.4	AD	het
14	PKD1	c.1997C>G	p.Ala666Gly	VUS	chr16	2165479	G	C	1	1560	0	NM_000296.4	AD	het
15	PKD1	c.7624G>T	p.Gly2542Cys	VUS	chr16	2156171	C	A	-	-	-	NM_001009944.3	AD	het
16	PKD1	c.5014_5015delAG	p.Arg1672Glyfs*98	Pathogenic	chr16	2160152	CCT	C	-	-	-	NM_000296.4	AD	het
17	SLC12A3	c.2521+253C>T	-	Pathogenic	chr16	56927219	C	T	-	-	-	NM_001126108.2	AR	het
18	SLC12A3#	c.1444-2A>G	-	VUS	chr16	56914040	A	G	-	-	-	NM_001126108.2	AR	het
19	WT1#	c.1276C>G	p.Leu426Val	VUS	chr11	32414239	G	C	-	-	-	NM_000378.6	AD	het
20	WT1#	c.1276C>G	p.Leu426Val	VUS	chr11	32414239	G	C	-	-	-	NM_000378.6	AD	het
21	WT1#	c.1276C>G	p.Leu426Val	VUS	chr11	32414239	G	C	-	-	-	NM_000378.6	AD	het
22	ATP6V0A4	c.1029+5G>A		Pathogenic	chr7	138437365	C	T	117	19938	0	NM_020632.3	AR	het
23	C3	c.1774C>T	p.Arg592Trp	Likely Pathogenic	chr19	6709766	G	A	0	18394	0	NM_000064.4	AD	het
24	COL4A3#	c.2024T>A	p.Ile675Asn	VUS	chr2	228142168	T	A	-	-	-	NM_000091.5	AD, AR	het
25	DNAJB11#	c.740+5_740+8del		VUS	chr3	186300560	CAAGT	C	0	15096	0	NM_016306.6	AD	het
26	INS	c.163C>T	p.Arg55Cys	Likely pathogenic	chr11	2182039	G	A	0	18378	0	NM_000207.3	AD	het
27	PAX2#	c.646_647del	p.Asp216Phefs*15	Likely pathogenic	chr10	102566213	GGA	G	-	-	-	NM_000278.5	AD	het
28	PIGA	c.298C>T	p.Gln100*	Likely	chrX	15349755	G	A	-	-	-	NM_002641.4	NA	Suspe

				pathogenic										cted chimerism
29	PKD1	c.1291C>T	p.Gln431*	Likely pathogenic	chr16	2167584	G	A	-	-	-	NM_001009944.3	AD	het
30	SCNN1B	c.1771C>T	p.Gln591*	VUS	chr16	23391970	C	T	-	-	-	NM_000336.3	AD	het
31	UMOD	c.113A>T	p.Asn38Ile	VUS	chr16	20360510	T	A	-	-	-	NM_003361.4	AD	het
32	UMOD	c.553C>T	p.Arg185Cys	Likely pathogenic	chr16	20360070	G	A	-	-	-	NM_003361.4	AD	het
33	UMOD	c.272_274del	p.Ser91del	Likely pathogenic	chr16	20360348	CAGG	C	-	-	-	NM_003361.4	AD	het
34	CUBN	c.10233G>A	p.Trp3411*	Pathogenic	chr10	16877142	C	T	2	19952	0	NM_001081.4	AR	het
35	CUBN	c.10233G>A	p.Trp3411*	Pathogenic	chr10	16877142	C	T	2	19952	0	NM_001081.4	AR	het
36	CUBN	c.10233G>A	p.Trp3411*	Pathogenic	chr10	16877142	C	T	2	19952	0	NM_001081.4	AR	het
37	CUBN	c.10233G>A	p.Trp3411*	Pathogenic	chr10	16877142	C	T	2	19952	0	NM_001081.4	AR	het
38	UMOD#	c.106C>T	p.H36Y	VUS	chr16	20360517	G	A	-	-	-	NM_003361.4	AD	het
39	UMOD#	c.106C>T	p.H36Y	VUS	chr16	20360517	G	A	-	-	-	NM_003361.4	AD	het
40	TSC1	c.250G>A	p.A84T	VUS	chr9	135801087	C	T	85	19938	1	NM_000368.5	AD	het
41	PLCE1#	c.1956-10C>T		VUS	chr10	95993801	C	T	21	19408	0	NM_016341.4	AR	het
42	PLCE1#	c.431T>C	p.L144P	VUS	chr10	95791234	T	C	-	-	-	NM_016341.4	AR	het
43	PKHD1#	c.6332+1_6332+2insG		Pathogenic	chr6	51777162	A	AC	-	-	-	NM_138694.4	AR	het
	PKHD1#	c.1187G>A	p.S396N	Likely Pathogenic	chr6	51924722	C	T	-	-	-	NM_138694.4	AR	het

	PKHD1	c.5935G>A	p.G1979R	Likely Pathogenic	chr6	51799094	C	T	-	-	-	NM_138694.4	AR	het
44	PKHD1	c.9529A>G	p.I3177V	Likely pathogenic	chr6	51612885	T	C	0	18388	0	NM_138694.4	AR	het
45	PKD2	c.637C>T	p.Arg213*	Pathogenic	chr4	88940651	C	T	0	1556	0	NM_000297.4	AD	het
46	PKD1	c.11710-2A>G	-	Likely pathogenic	chr16	2141177	T	C	-	-	-	NM_000296.4	AD	het
47	PKD1#	c.6280T>C	p.W2094R	Likely pathogenic	chr16	2158888	A	G	-	-	-	NM_00100944.3	AD	het
48	NPHP3	c.2694-2_2694-1delAG		Pathogenic	chr3	132408107	CCT	C	0	19952	0	NM_153240.5	AR	het
	NPHP3	c.1082C>G	p.S361C	VUS	chr3	132432006	G	C	61	18826	0	NM_153240.5	AR	het
49	CREBBP	c.1270C>T	p.Arg424*	Pathogenic	chr16	3842042	G	A	-	-	-	NM_004380.3	AD	het
50	COQ2	c.973A>G	p.T325A	VUS	chr4	84188867	T	C	9	12598	0	NM_015697	AR	het
51	COQ2	c.973A>G	p.T325A	VUS	chr4	84188867	T	C	9	12598	0	NM_015697	AR	het
52	COL4A4#	c.817-2A>G		Likely pathogenic	chr2	227967915	T	C	-	-	-	NM_000092.5	AR	het
53	COL4A3#	c.3391G>T	p.G1131C	VUS	chr2	228159259	G	T	-	-	-	NM_000091.5	AD, AR	het
54	CFB	c.2054_2057del	p.S687Pfs*16	Likely pathogenic	chr6	31919214	GGAGT	G	0	18394	0	NM_001710.6	AR	het
	COL4A1#	c.4988A>G	p.Q1663R	VUS	chr13	110802732	T	C	-	-	-	NM_001845.6	AD	het
55	ATP6V0A4	c.1534G>T	p.V512L	VUS	chr7	138424323	C	A	0	18392	0	NM_020632.3	AR	het

56	ATP6V0A4#	c.260C>A	p.T87N	VUS	chr7	138768811	G	T	-	-	-	NM_130841	AR	het
57	AGXT	c.823_824dupAG	p.S275Rfs*38	Pathogenic	chr2	241815390	T	TGA	3	18392	0	NM_000030.3	AR	het
	AGXT	c.595G>A	p.G199S	Likely Pathogenic	chr2	241812466	G	A	-	-	-	NM_000030.3	AR	het
58	ALG9#	c.1085G>C	p.W362S	VUS	chr11	111680406	C	G	-	-	-	NM_001352422	AD	het
59	ARL2BP#	c.397_398delGA	p.E133fs*38	Pathogenic	chr16	57286082	AAG	A	-	-	-	NM_012106.4	AR	hom
60	CFI	c.310G>A	p.G104R(p.Gly104Arg)	VUS	chr4	110687728	C	T	11	18394	0	NM_000204.5	AD	het
61	COL4A5	c.834+2T>G		Likely pathogenic	chrX	107823818	T	G	-	-	-	NM_000495.5	XL	het
62	GANAB	c.1078A>G	p.M360V	VUS	chr11	62398640	T	C	110	19920	0	NM_198335.4	AR	het
63	PKD1#	c.5661_5662insAGCACCAGGCCCAC	p.V1888fs*66	Likely pathogenic	chr16	2159506	C	CGTGGGCCTGGTGCT	-	-	-	NM_001009944.3	AD	het
64	PKD1	c.12178C>T	p.Q4060*	Likely pathogenic	chr16	2140552	G	A	-	-	-	NM_001009944.3	AD	het
65	PKD1	c.8147T>C	p.Ile2716Thr	VUS	chr16	2154513	A	G	-	-	-	NM_000296.4	AD	het
66	PKD1#	c.4590G>A	p.W1530*	Pathogenic	chr16	2160578	C	T	-	-	-	NM_001009944	AD	het
67	PKD1#	c.1357C>T	p.Gln453Ter	Pathogenic	chr16	2167518	G	A	-	-	-	NM_001009944	AD	het
68	PKD1	c.3931G>A	p.Ala1311Thr	Likely Pathogenic	chr16	2161237	C	T	322	19322	5	NM_001009944	AD	het

69	PKD2	c.2509G>T	p.E837*	Pathogenic	chr4	88989200	G	T	-	-	-	NM_000297	AD	het
70	PKD2	c.1837C>T	p.Q613*	Pathogenic	chr4	88977358	C	T	-	-	-	NM_000297.4	AD	het
71	PKD1	c.4636C>T	p.R1546W	VUS	chr16	2160532	G	A	3	19728	0	NM_001009944.3	AD	het
	PKD1	c.5228G>A	p.Arg1743Gln	VUS	chr16	2165510	G	C	-	-	-	NM_001009944.3	AD	het
72	TSC2	c.2032G>A	p.Ala678Thr	Likely pathogenic	chr16	2121870	G	A	238	17632	3	NM_000548.5	AD	het
	TSC2	c.5228G>A	p.Arg1743Gln	Likely pathogenic	chr16	2138295	G	A	0	18380	0	NM_000548.5	AD	het
73	UMOD#	c.93G>C	p.Trp31Cys	VUS	chr16	20360530	C	G	-	-	-	NM_003361.4	AD	het
74	WDR72	c.536C>G	p.Ser179*	Pathogenic	chr15	54006686	G	C	10	18388	0	NM_182758.4	AR	hom
75	COL4A4	c.4550T>G	p.Phe1517Cys	VUS	chr2	227872993	A	C	-	-	-	NM_000092.5	AR	het
	COL4A4	c.735+3A>G	-	VUS	chr2	227973294	T	C	-	-	-	NM_000092.5	AR	het
76	PKD2	c.958C>T	p.Arg320*	Pathogenic	chr4	88959517	C	T	1	30616	0	NM_000297.4	AD	het
77	COL4A3	c.3856G>A	p.Gly1286Ar	VUS	chr2	228163502	G	A	19	19532	0	NM_000091.5	AD	het
78	COL4A3	c.3856G>A	p.Gly1286Ar	VUS	chr2	228163502	G	A	19	19532	0	NM_000091.5	AD	het
79	PKD1	c.5086C>T	p.Gln1696*	Pathogenic	chr16	2160082	G	A	-	-	-	NM_001009944.3	AD	het
80	PKD1	c.11249G>A	p.Arg3750Gln	Likely pathogenic	chr16	2142501	C	T	0	18298	0	NM_001009944.3	AD	het
81	COL4A3#	c.2166delT	p.Pro723Leufs*24	Likely	chr2	22814454	GT	G	-	-	-	NM_000091.5	AD	het

				pathogenic		8								
82	COL4A3	c.4793T>G	p.Leu1598Arg	Pathogenic	chr2	228175529	T	G	15	19536	0	NM_000091.5	AD	het
83	PKD1	c.7113_7114delGT	p.Ser2372Leufs*47	Pathogenic	chr16	2156900	GAC	G	-	-	-	NM_001009944.3	AD	het
84	SLC12A3	c.1964G>A	p.Arg655His	Pathogenic	chr16	56920314	G	A	0	19946	0	NM_001126108.2	AR	het
85	COL4A3	c.4793T>G	p.Leu1598Arg	Pathogenic	chr2	228175529	T	G	15	19536	0	NM_000091.5	AD	het
86	PKD1#	c.322G>A	p.Glu108Lys	VUS	chr16	2169152	C	T	0	15072	0	NM_001009944.3	AD	het
87	AGXT	c.145A>C	p.Met49Leu	VUS	chr2	241808427	A	C	122	18910	0	NM_000030.3	AR	het
88	PKD1	c.3640G>T	p.Gly1214*	Pathogenic	chr16	2161528	C	A	-	-	-	NM_001009944.3	AD	het
89	PKD1	c.4274G>C	p.Arg1425Pro	Pathogenic	chr16	2160894	C	G	0	18362	0	NM_001009944.3	AD	het
90	PKD2#	c.1951delG	p.Glu651Lysfs*23	Pathogenic	chr4	88979185	AG	A	-	-	-	NM_000297.4	AD	het
91	PKD2#	c.1951delG	p.Glu651Lysfs*23	Pathogenic	chr4	88979185	AG	A	-	-	-	NM_000297.3	AD	het
92	PKD2#	c.1951delG	p.Glu651Lysfs*23	Pathogenic	chr4	88979185	AG	A	-	-	-	NM_000297.3	AD	het
93	PKD2#	c.1951delG	p.Glu651Lysfs*23	Pathogenic	chr4	88979185	AG	A	-	-	-	NM_000297.3	AD	het
94	DAAM2	c.1004G>A	p.Arg335Gln	Likely pathogenic	chr6	39839308	G	A	0	12524	0	NM_001201427.2	AR	het
95	PKD1	c.1297C>T	p.Gln433*	Pathogenic	chr16	2167578	G	A	-	-	-	NM_001009944.3	AD	het
96	PKD1	c.1297C>T	p.Gln433*	Pathogenic	chr16	2167578	G	A	-	-	-	NM_001009944.	AD	het

												3		
97	PKD1	c.4306C>T	p.Arg1436*	Pathogenic	chr16	2160862	G	A	-	-	-	NM_001009944.3	AD	het
98	PKD1	c.1297C>T	p.Gln433*	Pathogenic	chr16	2167578	G	A	-	-	-	NM_001009944.3	AD	het
99	COL4A4	c.4421C>T	p.Thr1474Met	Likely pathogenic	chr2	227875130	G	A	34	19530	0	NM_000092.5	AR	het
100	PKD2	c.973C>T	p.Arg325*	Pathogenic	chr4	88959532	C	T	0	18382	0	NM_000297.4	AR	het
101	PKD2	c.973C>T	p.Arg325*	Pathogenic	chr4	88959532	C	T	0	18382	0	NM_000297.4	AD	het
102	PKD2	c.973C>T	p.Arg325*	Pathogenic	chr4	88959532	C	T	0	18382	0	NM_000297.4	AD	het
103	SLC5A2	c.409C>T	p.Arg137Cys	VUS	chr16	31497155	C	T	0	19952	0	NM_003041.4	AD	het
104	CFH	c.3643C>G	p.Arg1215Gly	Likely pathogenic	chr1	196716390	C	G	-	-	-	NM_000186.4	AD	het
105	CLCNKB	1p36.13 homozygous deletion		Pathogenic	chrX	100653052	TGA	T	-	-	-	NM_000169.3	XL	hem
106	COL4A5	chrX:107801949-107830693	exon3-19 deletion		chrX				-	-	-		XL	hem
107	CFH		exon 22 chimeric deletion		chrX				-	-	-		AD	mosaic
108	COL4A5	chrX:g.107819139-107819203del exon10 deletion		VUS	chrX				-	-	-		XL	het

Note: All population frequency data are derived from gnomAD v2.1.1 (GRCh37). For patient 11 with a COL4A5 variant, the hemizygote allele frequency is 36. #indicates that this variant site is a de novo variant site. AD, autosomal dominant inheritance; AR, autosomal recessive inheritance; XL, X-linked recessive inheritance; NA, not available; Het, heterozygote; Hom, homozygote; Hem, hemizygote.

Supplemental Table 2: The variation frequency of each variant within the cohort and the variation frequency data retrieved from mainstream databases.

Gene	HGVS		Variation frequency	gnomAD_genomes_EAS_AF	gnomAD_exomes_EAS_AF	gnomAD_genomes_AF	gnomAD_exomes_AF	1000Gp3_AF	1000Gp3_EAS_AF	gnomAD_genomes_POPMAX_AF
GLA	c.1033_1034delTC	p.Ser345Argfs*29	1/580	-	-	-	-	-	-	-
PKD1	c.6915_6915+2delGGT	-	1/580	-	-	-	-	-	-	-
KCNJ16	c.689G>A	p.Arg230Lys	1/580	2.699580e-03	1.963990e-03	1.314370e-04	2.502280e-04	7.9872204472843445E-4	0.00297619047619047 6	2.699580e-03
AGXT	c.823_824dupAG	p.Ser275Argfs*38	1/580	-	-	-	-	-	-	-
AGXT	c.638C>T	p.Ala213Val	1/580	3.859510e-04	2.720050e-04	1.313680e-05	1.990860e-05	1.9968051118210862E-4	9.9206349206349 2E-4	3.859510e-04
CFB	c.121G>C	p.Gly41Arg	2/580	-	-	-	-	-	-	-
CFB	c.121G>C	p.Gly41Arg	2/580	-	-	-	-	-	-	-
CFHR3	c.613+90A>C	-	1/580	-	-	-	-	-	-	-
CLCNKB	c.280C>A	p.Leu94Ile	1/580	3.658070e-03	4.077860e-03	1.445690e-04	3.102500e-04	0.0011980830670926517	0.00595238095238095 2	3.658070e-03
COL4A3	c.1909G>A	p.Gly637Arg	1/580	0.000000e+00	5.576000e-05	6.572630e-06	8.126320e-06	-	-	1.470240e-05
COL4A4	c.1786G>T	p.Gly596Trp	1/580	-	-	-	-	-	-	-
COL4A5	c.2215C>G	p.Pro739Ala	1/580	1.734930e-02	1.343540e-02	6.048570e-04	1.155070e-03	0.003443708609271523	0.017015706806282723	1.734930e-02
NPHS1	c.3478C>T	p.Arg1160*	1/580	0.000000e+00	5.436560e-05	6.573330e-06	9.942970e-05	-	-	1.470070e-05
PKD1	c.1997C>G	p.Ala666Gly	2/580	3.865480e-04	-	1.314790e-05	-	-	-	3.865480e-04
PKD1	c.1997C>G	p.Ala666Gly	2/580	3.865480e-04	-	1.314790e-05	-	-	-	3.865480e-04
PKD1	c.7624G>T	p.Gly2542Cys	1/580	-	-	-	-	-	-	-
PKD1	c.5014_5	p.Arg167	1/580	-	-	-	-	-	-	-

	015delAG	2Glyfs*98								
SLC12A3	c.2521+253C>T	-	1/580	-	-	-	-	-	-	-
SLC12A3	c.1444-2A>G	-	1/580	-	-	-	-	-	-	-
WT1	c.1276C>G	p.Leu426Val	3/580	-	-	-	-	-	-	-
WT1	c.1276C>G	p.Leu426Val	3/580	-	-	-	-	-	-	-
WT1	c.1276C>G	p.Leu426Val	3/580	-	-	-	-	-	-	-
ATP6V0A4	c.1029+5G>A		1/580	-	-	-	-	-	-	-
C3	c.1774C>T	p.Arg592Trp	1/580	-	0.000000e+00	-	3.976710e-06	-	-	-
COL4A3	c.2024T>A	p.Ile675Asn	1/580	-	-	-	-	-	-	-
DNAJB11	c.740+5_740+8del		1/580	-	-	-	-	-	-	-
INS	c.163C>T	p.Arg55Cys	1/580	-	-	-	-	-	-	-
PAX2	c.646_647del	p.Asp216Phefs*15	1/580	-	-	-	-	-	-	-
PIGA	c.298C>T	p.Gln100*	1/580	-	-	-	-	-	-	-
PKD1	c.1291C>T	p.Gln431*	1/580	-	-	-	-	-	-	-
SCNN1B	c.1771C>T	p.Gln591*	1/580	-	-	-	-	-	-	-
UMOD	c.113A>T	p.Asn38Ile	1/580	-	-	-	-	-	-	-
UMOD	c.553C>T	p.Arg185Cys	1/580	-	-	-	-	-	-	-
UMOD	c.272_274del	p.Ser91del	1/580	-	-	-	-	-	-	-
CUBN	c.10233G>A	p.Trp3411*	4/580	1.921600e-04	5.437150e-05	1.972050e-05	1.193690e-05	-	-	1.921600e-04
CUBN	c.10233G>A	p.Trp3411*	4/580	1.921600e-04	5.437150e-05	1.972050e-05	1.193690e-05	-	-	1.921600e-04
CUBN	c.10233G>A	p.Trp3411*	4/580	1.921600e-04	5.437150e-05	1.972050e-05	1.193690e-05	-	-	1.921600e-04
CUBN	c.10233G>A	p.Trp3411*	4/580	1.921600e-04	5.437150e-05	1.972050e-05	1.193690e-05	-	-	1.921600e-04
UMOD	c.106C>T	p.H36Y	2/580	-	-	-	-	-	-	-
UMOD	c.106C>T	p.H36Y	2/580	-	-	-	-	-	-	-

TSC1	c.250G>A	p.A84T	1/580	5.583370e-03	4.080970e-03	3.680000e-04	7.172230e-04	0.0013977635782747603	0.00694444444444444	5.583370e-03
PLCE1	c.1956-10C>T		1/580	-	-	-	-	-	-	-
PLCE1	c.431T>C	p.L144P	1/580	-	-	-	-	-	-	-
PKHD1	c.6332+1_6332+2insG		1/580	-	-	-	-	-	-	-
PKHD1	c.1187G>A	p.S396N	1/580	-	-	-	-	-	-	-
PKHD1	c.5935G>A	p.G1979R	1/580	-	-	-	-	-	-	-
PKHD1	c.9529A>G	p.I3177V	1/580	0.000000e+00	0.000000e+00	1.314250e-05	1.594500e-05	-	-	6.545360e-05
PKD2	c.637C>T	p.Arg213*	1/580	0.000000e+00	-	6.580420e-06	-	-	-	2.417680e-05
PKD1	c.11710-2A>G	-	1/580	-	-	-	-	-	-	-
PKD1	c.6280T>C	p.W2094R	1/580	-	-	-	-	-	-	-
NPHP3	c.2694-2_2694-1delAG		1/580	-	-	-	-	-	-	-
NPHP3	c.1082C>G	p.S361C	1/580	3.465540e-03	3.069260e-03	1.184800e-04	2.401370e-04	7.987220447284345E-4	0.003968253968253968	3.465540e-03
CREBBP	c.1270C>T	p.Arg424*	1/580	-	-	-	-	-	-	-
COQ2	c.973A>G	p.T325A	2/580	5.784810e-04	6.340580e-04	1.971350e-05	5.607060e-05	-	-	5.784810e-04
COQ2	c.973A>G	p.T325A	2/580	5.784810e-04	6.340580e-04	1.971350e-05	5.607060e-05	-	-	5.784810e-04
COL4A4	c.817-2A>G		1/580	-	-	-	-	-	-	-
COL4A3	c.3391G>T	p.G1131C	1/580	-	-	-	-	-	-	-
CFB	c.2054_2057del	p.S687Pfs*16	1/580	-	-	-	-	-	-	-
COL4A1	c.4988A>G	p.Q1663R	1/580	-	-	-	-	-	-	-
ATP6V0A4	c.1534G>T	p.V512L	1/580	-	0.000000e+00	-	7.955260e-06	-	-	-
ATP6V0	c.260C>A	p.T87N	1/580	-	-	-	-	-	-	-

A4										
AGXT	c.823_824dupAG	p.S275Rfs*38	1/580	-	-	-	-	-	-	-
AGXT	c.595G>A	p.G199S	1/580	-	-	-	-	-	-	-
ALG9	c.1085G>C	p.W362S	1/580	-	-	-	-	-	-	-
ARL2BP	c.397_398delGA	p.E133fs*38	1/580	-	-	-	-	-	-	-
CFI	c.310G>A	p.G104R(p.Gly104Arg)	1/580	5.773670e-04	5.980210e-04	1.971840e-05	5.574450e-05	5.990415335463259E-4	0.00297619047619047 6	5.773670e-04
COL4A5	c.834+2T>G		1/580	-	-	-	-	-	-	-
GANAB	c.1078A>G	p.M360V	1/580	5.226480e-03	5.282650e-03	1.841140e-04	4.131700e-04	0.001198083067092651 7	0.0049603174603174 6	5.226480e-03
PKD1	c.5661_5662insAGCACCAGGCCCAC	p.V1888fs*66	1/580	-	-	-	-	-	-	-
PKD1	c.12178C>T	p.Q4060*	1/580	-	-	-	-	-	-	-
PKD1	c.8147T>C	p.Ile2716Thr	1/580	0.000000e+00	-	6.722330e-06	-	-	-	2.520160e-05
PKD1	c.4590G>A	p.W1530*	1/580	-	-	-	-	-	-	-
PKD1	c.1357C>T	p.Gln453Ter	1/580	-	-	-	-	-	-	-
PKD1	c.3931G>A	p.Ala1311Thr	1/580	1.680190e-02	1.610000e-02	6.045150e-04	1.221520e-03	0.001397763578274760 3	0.00694444444444444 4	1.680190e-02
PKD2	c.2509G>T	p.E837*	1/580	-	-	-	-	-	-	-
PKD2	c.1837C>T	p.Q613*	1/580	-	-	-	-	-	-	-
PKD1	c.4636C>T	p.R1546W	1/580	0.000000e+00	1.651070e-04	3.088890e-04	1.251870e-04	-	-	1.013710e-03
PKD1	c.5228G>A	p.Arg1743Gln	1/580	3.856540e-04	-	1.314460e-05	-	-	-	3.856540e-04
TSC2	c.2032G>A	p.Ala678Thr	1/580	1.272660e-02	1.325120e-02	6.635130e-04	1.109530e-03	0.002196485623003195	0.01091269841269841 2	1.272660e-02
TSC2	c.5228G>A	p.Arg1743Gln	1/580	-	-	-	-	-	-	-

UMOD	c.93G>C	p.Trp31Cys	1/580	-	-	-	-	-	-	-
WDR72	c.536C>G	p.Ser179*	1/580	1.931250e-04	5.438330e-04	6.578510e-06	3.981750e-05	1.9968051118210862E-4	9.9206349206349 2E-4	1.931250e-04
COL4A4	c.4550T>G	p.Phe1517Cys	1/580	-	-	-	-	-	-	-
COL4A4	c.735+3A>G	-	1/580	-	-	-	-	-	-	-
PKD2	c.958C>T	p.Arg320*	1/580	0.000000e+00	0.000000e+00	1.314350e-05	3.981870e-06	-	-	4.825320e-05
COL4A3	c.3856G>A	p.Gly1286Ar	2/580	5.782580e-04	8.902740e-04	1.971760e-05	6.420190e-05	-	-	5.782580e-04
COL4A3	c.3856G>A	p.Gly1286Ar	2/580	5.782580e-04	8.902740e-04	1.971760e-05	6.420190e-05	-	-	5.782580e-04
PKD1	c.5086C>T	p.Gln1696*	1/580	-	-	-	-	-	-	-
PKD1	c.11249G>A	p.Arg3750Gln	1/580	-	0.000000e+00	-	4.044420e-06	-	-	-
COL4A3	c.2166delT	p.Pro723Leufs*24	1/580	-	-	-	-	-	-	-
COL4A4	c.4793T>G	p.Leu1598Arg	2/580	9.622790e-04	7.787300e-04	3.284630e-05	5.612660e-05	-	-	9.622790e-04
PKD1	c.7113_7114delGT	p.Ser2372Leufs*47	1/580	-	-	-	-	-	-	-
SLC12A3	c.1964G>A	p.Arg655His	1/580	0.000000e+00	0.000000e+00	9.858690e-05	7.967370e-05	-	-	1.447670e-04
COL4A3	c.4793T>G	p.Leu1598Arg	2/580	9.622790e-04	7.787300e-04	3.284630e-05	5.612660e-05	-	-	9.622790e-04
PKD1	c.322G>A	p.Glu108Lys	1/580	-	-	-	-	-	-	-
AGXT	c.145A>C	p.Met49Leu	1/580	8.185840e-03	6.182730e-03	3.020150e-04	4.976590e-04	0.001996805111821086	0.0099206349206349 2	8.185840e-03
PKD1	c.3640G>T	p.Gly1214*	1/580	-	-	-	-	-	-	-
PKD1	c.4274G>C	p.Arg1425Pro	1/580	0.000000e+00	0.000000e+00	6.571170e-06	4.017000e-06	-	-	2.411850e-05
PKD2	c.1951delG	p.Glu651Lysfs*23	4/580	-	-	-	-	-	-	-
PKD2	c.1951delG	p.Glu651Lysfs*23	4/580	-	-	-	-	-	-	-
PKD2	c.1951delG	p.Glu651Lysfs*23	4/580	-	-	-	-	-	-	-

PKD2	c.1951delG	p.Glu651Lysfs*23	4/580	-	-	-	-	-	-	-
DAAM2	c.1004G>A	p.Arg335Gln	1/580	0.000000e+00	0.000000e+00	3.287310e-04	3.126640e-04	1.9968051118210862E-4	0.0	6.320560e-04
PKD1	c.1297C>T	p.Gln433*	3/580	-	-	-	-	-	-	-
PKD1	c.1297C>T	p.Gln433*	3/580	-	-	-	-	-	-	-
PKD1	c.4306C>T	p.Arg1436*	1/580	-	-	-	-	-	-	-
PKD1	c.1297C>T	p.Gln433*	3/580	-	-	-	-	-	-	-
COL4A4	c.4421C>T	p.Thr1474Met	1/580	1.734100e-03	1.669260e-03	7.226000e-05	1.484100e-04	3.9936102236421724E-4	0.001984126984126984	1.734100e-03
PKD2	c.973C>T	p.Arg325*	3/580	1.926780e-04	0.000000e+00	1.314800e-05	1.194750e-05	-	-	1.926780e-04
PKD2	c.973C>T	p.Arg325*	3/580	1.926780e-04	0.000000e+00	1.314800e-05	1.194750e-05	-	-	1.926780e-04
PKD2	c.973C>T	p.Arg325*	3/580	1.926780e-04	0.000000e+00	1.314800e-05	1.194750e-05	-	-	1.926780e-04
SLC5A2	c.409C>T	p.Arg137Cys	1/580	0.000000e+00	0.000000e+00	3.284850e-05	2.392840e-05	-	-	1.307870e-04
CFH	c.3643C>G	p.Arg1215Gly	1/580	-	-	-	-	-	-	-
COL4A5	chrX:107801949-107830693	exon3-19 deletion		-	-	-	-	-	-	-
CFH	exon 22 chimeric deletion			-	-	-	-	-	-	-
COL4A5	chrX:g.107819139-107819203del	exon10 deletion		-	-	-	-	-	-	-
