# Supplementary material for: Prevalence and Genetic Spectrum of Inherited Kidney Diseases: A Chinese Cohort Study
Source: Kidney360. 2026 Apr 1;7(7):1615–24. doi: 10.34067/KID.0000001183 (PMC13450970; doi:10.34067/KID.0000001183)
Supplement: Supplementary file 2 [file kidney360-7-1615-s002.pdf]

ASN Journal Disclosure Form

As per ASN journal policy, I have disclosed any financial relationships or commitments I have held in the past 36 months as included below. I have listed my Current Employer below to indicate there is a relationship requiring disclosure. If no relationship exists, my Current Employer is not listed.

A. Aierken has nothing to disclose.

I understand that the information above will be published within the journal article, if accepted, and that failure to comply and/or to accurately and completely report the potential financial conflicts of interest could lead to the following: 1) Prior to publication, article rejection, or 2) Post-publication, sanctions ranging from, but not limited to, issuing a correction, reporting the inaccurate information to the authors' institution, banning authors from submitting work to ASN journals for varying lengths of time, and/or retraction of the published work.

Name: Ailima Aierken
Manuscript ID: K360-2025-000836R2
Manuscript Title: Prevalence and Genetic Spectrum of Inherited Kidney Diseases in China
Date of Completion: January 4, 2026
Disclosure Updated Date: January 4, 2026

ASN American Society of Nephrology | JASN Journal of the American Society of Nephrology | KIDNEY360 Accessing Our World From Every Angle | CJASN Clinical Journal of the American Society of Nephrology

ASN Journal Disclosure Form

As per ASN journal policy, I have disclosed any financial relationships or commitments I have held in the past 36 months as included below. I have listed my Current Employer below to indicate there is a relationship requiring disclosure. If no relationship exists, my Current Employer is not listed.

H. Jiang has nothing to disclose.

I understand that the information above will be published within the journal article, if accepted, and that failure to comply and/or to accurately and completely report the potential financial conflicts of interest could lead to the following: 1) Prior to publication, article rejection, or 2) Post-publication, sanctions ranging from, but not limited to, issuing a correction, reporting the inaccurate information to the authors' institution, banning authors from submitting work to ASN journals for varying lengths of time, and/or retraction of the published work.

Name: Hong Jiang
Manuscript ID: K360-2025-000836R2
Manuscript Title: Prevalence and Genetic Spectrum of Inherited Kidney Diseases in China
Date of Completion: January 4, 2026
Disclosure Updated Date: January 4, 2026

ASN American Society of Nephrology
JASN Journal of the American Society of Nephrology
KIDNEY360 Accessing Our World From Every Angle
CJASN Clinical Journal of the American Society of Nephrology

ASN Journal Disclosure Form

As per ASN journal policy, I have disclosed any financial relationships or commitments I have held in the past 36 months as included below. I have listed my Current Employer below to indicate there is a relationship requiring disclosure. If no relationship exists, my Current Employer is not listed.

Z. Li reports the following:
Employer: Zhong Da Hospital, Southeast University,

I understand that the information above will be published within the journal article, if accepted, and that failure to comply and/or to accurately and completely report the potential financial conflicts of interest could lead to the following: 1) Prior to publication, article rejection, or 2) Post-publication, sanctions ranging from, but not limited to, issuing a correction, reporting the inaccurate information to the authors' institution, banning authors from submitting work to ASN journals for varying lengths of time, and/or retraction of the published work.

Name: Zuolin Li
Manuscript ID: K360-2025-000836R4
Manuscript Title: Prevalence and Genetic Spectrum of Inherited Kidney Diseases in China
Date of Completion: March 3, 2026
Disclosure Updated Date: March 3, 2026

ASN American Society of Nephrology · JASN Journal of the American Society of Nephrology · KIDNEY360 Accessing Our World From Every Angle · CJASN Clinical Journal of the American Society of Nephrology

ASN Journal Disclosure Form

As per ASN journal policy, I have disclosed any financial relationships or commitments I have held in the past 36 months as included below. I have listed my Current Employer below to indicate there is a relationship requiring disclosure. If no relationship exists, my Current Employer is not listed.

Z. Liu has nothing to disclose.

I understand that the information above will be published within the journal article, if accepted, and that failure to comply and/or to accurately and completely report the potential financial conflicts of interest could lead to the following: 1) Prior to publication, article rejection, or 2) Post-publication, sanctions ranging from, but not limited to, issuing a correction, reporting the inaccurate information to the authors' institution, banning authors from submitting work to ASN journals for varying lengths of time, and/or retraction of the published work.

Name: Ziqi Liu
Manuscript ID: K360-2025-000836R1
Manuscript Title: Prevalence and Genetic Spectrum of Inherited Kidney Diseases in China
Date of Completion: November 27, 2025
Disclosure Updated Date: November 27, 2025

ASN
American Society of Nephrology
JASN
Journal of the American Society of Nephrology
KIDNEY360
Accessing Our World From Every Angle
CJASN
Clinical Journal of the American Society of Nephrology

ASN Journal Disclosure Form

As per ASN journal policy, I have disclosed any financial relationships or commitments I have held in the past 36 months as included below. I have listed my Current Employer below to indicate there is a relationship requiring disclosure. If no relationship exists, my Current Employer is not listed.

Y. Tu has nothing to disclose.

I understand that the information above will be published within the journal article, if accepted, and that failure to comply and/or to accurately and completely report the potential financial conflicts of interest could lead to the following: 1) Prior to publication, article rejection, or 2) Post-publication, sanctions ranging from, but not limited to, issuing a correction, reporting the inaccurate information to the authors' institution, banning authors from submitting work to ASN journals for varying lengths of time, and/or retraction of the published work.

Name: Yan Tu
Manuscript ID: K360-2025-000836R2
Manuscript Title: Prevalence and Genetic Spectrum of Inherited Kidney Diseases in China
Date of Completion: January 4, 2026
Disclosure Updated Date: January 4, 2026

ASN American Society of Nephrology
JASN Journal of the American Society of Nephrology
KIDNEY360 Accessing Our World From Every Angle
CJASN Clinical Journal of the American Society of Nephrology

ASN Journal Disclosure Form

As per ASN journal policy, I have disclosed any financial relationships or commitments I have held in the past 36 months as included below. I have listed my Current Employer below to indicate there is a relationship requiring disclosure. If no relationship exists, my Current Employer is not listed.

B. Wang reports the following:
Employer: Zhong Da Hospital, Southeast University

I understand that the information above will be published within the journal article, if accepted, and that failure to comply and/or to accurately and completely report the potential financial conflicts of interest could lead to the following: 1) Prior to publication, article rejection, or 2) Post-publication, sanctions ranging from, but not limited to, issuing a correction, reporting the inaccurate information to the authors' institution, banning authors from submitting work to ASN journals for varying lengths of time, and/or retraction of the published work.

Name: Bin Wang
Manuscript ID: K360-2025-000836R2
Manuscript Title: Prevalence and Genetic Spectrum of Inherited Kidney Diseases in China
Date of Completion: January 6, 2026
Disclosure Updated Date: January 6, 2026

ASN Journal Disclosure Form

As per ASN journal policy, I have disclosed any financial relationships or commitments I have held in the past 36 months as included below. I have listed my Current Employer below to indicate there is a relationship requiring disclosure. If no relationship exists, my Current Employer is not listed.

F. Wang reports the following:
Employer: Institute of Nephrology, Zhong Da Hospital, Southeast University School of Medicine

I understand that the information above will be published within the journal article, if accepted, and that failure to comply and/or to accurately and completely report the potential financial conflicts of interest could lead to the following: 1) Prior to publication, article rejection, or 2) Post-publication, sanctions ranging from, but not limited to, issuing a correction, reporting the inaccurate information to the authors' institution, banning authors from submitting work to ASN journals for varying lengths of time, and/or retraction of the published work.

Name: Fengmei Wang
Manuscript ID: K360-2025-000836R4
Manuscript Title: Prevalence and Genetic Spectrum of Inherited Kidney Diseases in China.
Date of Completion: March 2, 2026
Disclosure Updated Date: March 2, 2026

ASN
American Society of Nephrology
JASN
Journal of the American Society of Nephrology
KIDNEY360
Accessing Our World From Every Angle
CJASN
Clinical Journal of the American Society of Nephrology

ASN Journal Disclosure Form

As per ASN journal policy, I have disclosed any financial relationships or commitments I have held in the past 36 months as included below. I have listed my Current Employer below to indicate there is a relationship requiring disclosure. If no relationship exists, my Current Employer is not listed.

Y. Yang has nothing to disclose.

I understand that the information above will be published within the journal article, if accepted, and that failure to comply and/or to accurately and completely report the potential financial conflicts of interest could lead to the following: 1) Prior to publication, article rejection, or 2) Post-publication, sanctions ranging from, but not limited to, issuing a correction, reporting the inaccurate information to the authors' institution, banning authors from submitting work to ASN journals for varying lengths of time, and/or retraction of the published work.

Name: Yan Yang
Manuscript ID: K360-2025-000836R2
Manuscript Title: Prevalence and Genetic Spectrum of Inherited Kidney Diseases in China
Date of Completion: January 5, 2026
Disclosure Updated Date: January 5, 2026

ASN American Society of Nephrology
JASN Journal of the American Society of Nephrology
KIDNEY360 Accessing Our World From Every Angle
CJASN Clinical Journal of the American Society of Nephrology

ASN Journal Disclosure Form

As per ASN journal policy, I have disclosed any financial relationships or commitments I have held in the past 36 months as included below. I have listed my Current Employer below to indicate there is a relationship requiring disclosure. If no relationship exists, my Current Employer is not listed.

Q. Yin reports the following:
Employer: Southeast University

I understand that the information above will be published within the journal article, if accepted, and that failure to comply and/or to accurately and completely report the potential financial conflicts of interest could lead to the following: 1) Prior to publication, article rejection, or 2) Post-publication, sanctions ranging from, but not limited to, issuing a correction, reporting the inaccurate information to the authors' institution, banning authors from submitting work to ASN journals for varying lengths of time, and/or retraction of the published work.

Name: Qing Yin
Manuscript ID: K360-2025-000836R4
Manuscript Title: Prevalence and Genetic Spectrum of Inherited Kidney Diseases in China
Date of Completion: March 3, 2026
Disclosure Updated Date: March 3, 2026

ASN
American Society of Nephrology
JASN
Journal of the American Society of Nephrology
KIDNEY360
Accessing Our World From Every Angle
CJASN
Clinical Journal of the American Society of Nephrology

ASN Journal Disclosure Form

As per ASN journal policy, I have disclosed any financial relationships or commitments I have held in the past 36 months as included below. I have listed my Current Employer below to indicate there is a relationship requiring disclosure. If no relationship exists, my Current Employer is not listed.

J. Zhuang has nothing to disclose.

I understand that the information above will be published within the journal article, if accepted, and that failure to comply and/or to accurately and completely report the potential financial conflicts of interest could lead to the following: 1) Prior to publication, article rejection, or 2) Post-publication, sanctions ranging from, but not limited to, issuing a correction, reporting the inaccurate information to the authors' institution, banning authors from submitting work to ASN journals for varying lengths of time, and/or retraction of the published work.

Name: Jing Zhuang
Manuscript ID: K360-2025-000836R4
Manuscript Title: Prevalence and Genetic Spectrum of Inherited Kidney Diseases in China.
Date of Completion: March 3, 2026
Disclosure Updated Date: March 3, 2026
